# Simultaneous existence of acute myeloid leukemia and chronic lymphocytic leukemia: a case report

**DOI:** 10.1186/s12885-016-2780-5

**Published:** 2016-09-19

**Authors:** Eman Al Mussaed, Hani Osman, Ghaleb Elyamany

**Affiliations:** 1 Department of Basic Sciences, Hematopathology Division, Princess Nourah Bint Abdulrahman University, College of Medicine, Riyadh, Saudi Arabia; 2 Department of Adult Clinical Hematology and Stem cell Therapy, Prince Sultan Military Medical City, Riyadh, Kingdom of Saudi Arabia; 3 Department of Central Military Laboratory and Blood Bank, Prince Sultan Military Medical City, PO Box 7897, Riyadh, 11159 Kingdom of Saudi Arabia

**Keywords:** Chronic lymphocytic leukemia, Acute myeloid leukemia, Case rep

## Abstract

**Background:**

The simultaneous Occurrence of chronic lymphocytic leukemia (CLL) and acute myeloid leukemia (AML) has been rarely reported. Most of these cases have been occurring more frequently as a secondary event in patients receiving chemotherapeutic agents for CLL.

**Case presentation:**

We describe a case of a 77-year-old man who presented with fatigue, pallor and lower limb pain and weakness. Initial laboratory studies showed Hb 7.7 g/dl, WBC 279.6 × 10^9^/1, PLT 143× 10^9^/1. The peripheral blood (PB) smear examination showed circulating blast cells (20 %) cells and 50 % lymphocytes, with smudge cells.

A bone marrow examination showed infiltration by two discrete abnormal cell populations, one represents the leukemic blast cells (60 %) and the other represents small mature lymphocytes (30 %). The immunologic phenotype of blasts was characterized by the co-expression of CD13, CD33, CD14, CD4, CD15, CD64, HLA-DR, CD11c. Lymphocytes were characterized by a typical CLL immunophenotype: CD19+, CD5+, CD23+, CD20+ (dim) and negative for FMC7, CD34, CD10 and TdT. Cytogenetic studies were negative for CLL and AML panels. PCR assays for AML specific genetic abnormalities were negative. Immunoglobulin gene analysis established the clonal nature of the B-cell expansion. A final diagnosis of concomitant CLL and AML(FAB: M5) was made.

**Conclusion:**

We have reported a case in which there was simultaneous presentation of AML and CLL. Both forms of leukemia were well documented by morphology, cytometric analysis and molecular studies. Our findings support the idea that this rare concurrence of AML and untreated CLL may represent two separate disease processes.

## Background

The association of different malignancies with chronic lymphocytic leukemia (CLL) such as lung and skin cancer has been reported [1–5]. The coexistence of acute myeloid leukemia (AML) and CLL in the same patient has been occasionally reported. Most of these cases have been reported to occur after treatment of CLL with cytotoxic drugs suggesting that AML may be a secondary leukemia [3, 6–8]. Cases in the absence of prior treatment are exceedingly rare. Only a minority of reports represent de novo AML following untreated CLL or concomitant AML and CLL appearing as two distinct and unrelated malignancies [4, 6, 9–12].

We report a case of concomitant CLL and AML (FAB: M5) without previous exposure to a cytotoxic agent or irradiation. Morphologic features, flow cytometric analysis, molecular and cytogenetic findings of peripheral blood and Bone marrow samples are discussed.

## Case presentation

A 77 year-old man presented with 3 weeks history of fatigue, pallor and lower limb pain and weakness with no history of previous exposure to a cytotoxic agent, irradiation or other medications except for hypertension. There was no history of fever, night sweats, weight loss, anorexia, chest pain, headache, blurring of vision or other relevant symptoms.

Physical examination was significant for enlarged spleen (3 cm below the costal margin), but no lymphadenopathy, hepatomegaly, jaundice or gum hypertrophy. Chest X-ray was normaland a computerized tomography (CT) scan revealed no lymphoadenopathies.

**Fig. 1:**
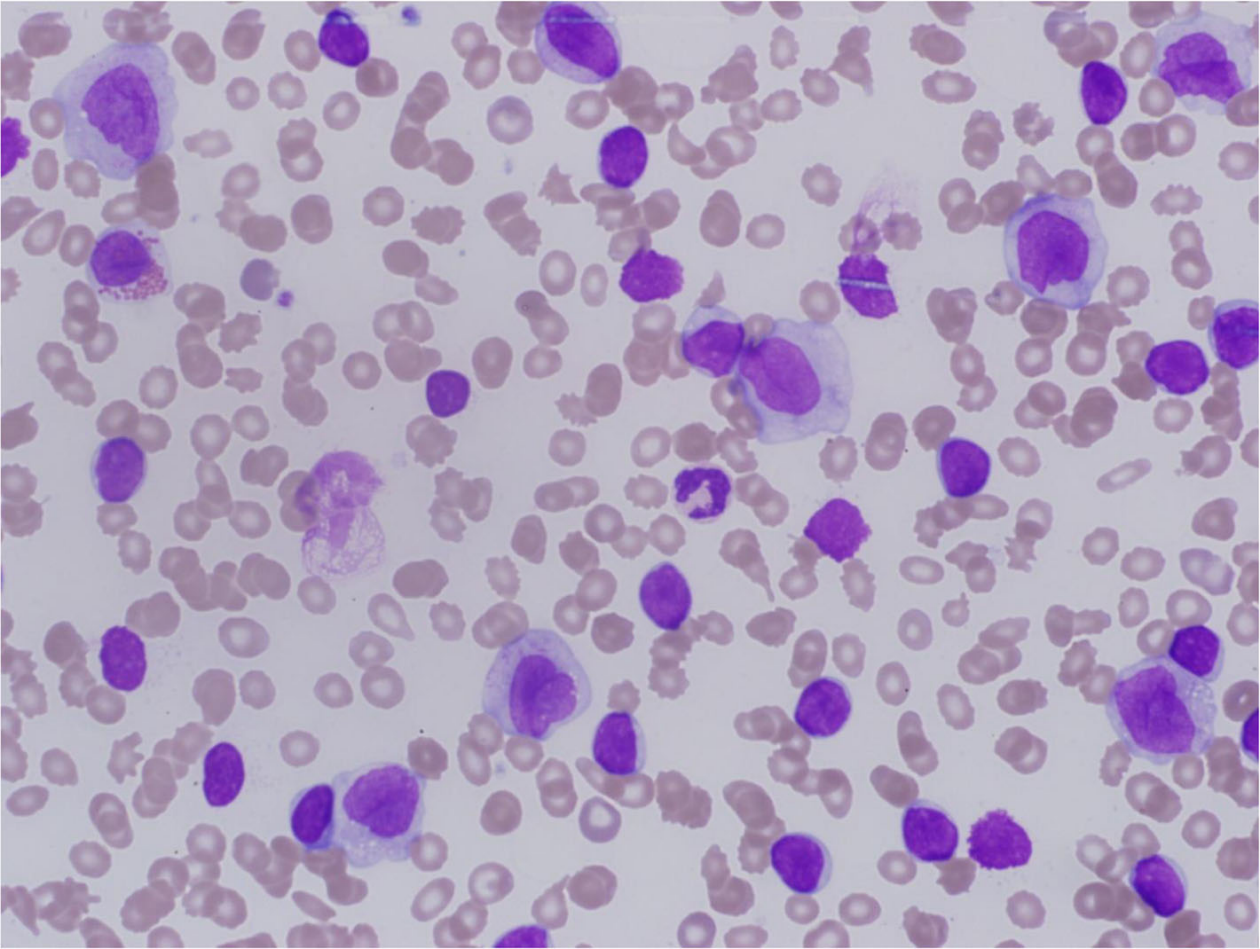
PB smear showing circulating blast cells with monocytoid featuresand small mature looking lymphocytes with smudge cell

Initial PB count and smears showed anemia, thrombocytopenia, and leukocytosis (hemoglobin: 7.7 g/dl, white blood cells (WBC) 279.6 × 10^9^/1, platelets 45 × 10^9^/1) with two distinct abnormal populations, the small cells were corresponding to mature lymphocytes (60 %) and large cells which corresponded to blast cells (20 %), and smear cells were also present (Fig. 1).

**Fig. 2:**
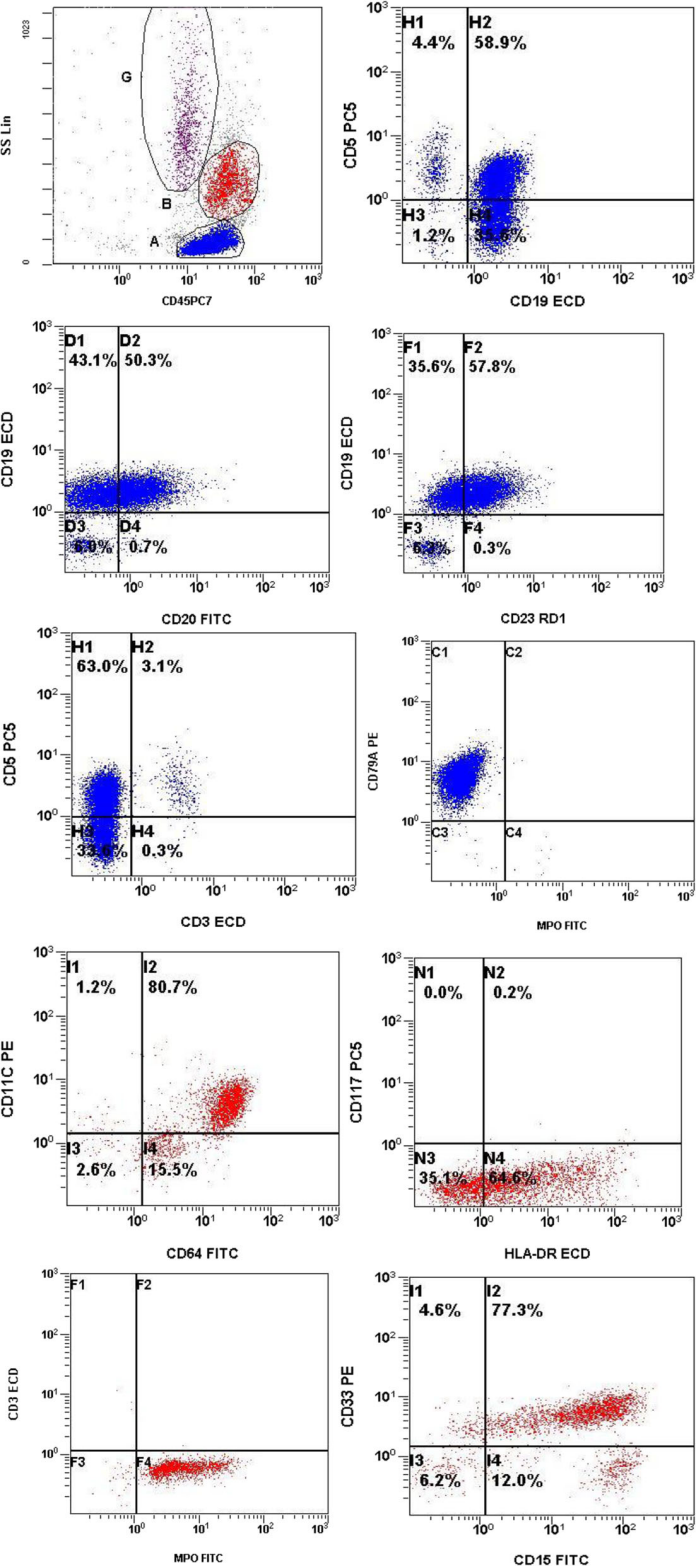
Immunophenotyping on Peripheral Blood sample. Lymphocytes (blue color) were characterized by a typicall CLL immunophenotype: CD19+, CD5+, CD23+, CD20 + (dim), CD79a + and negative for CD3 whereas the immunologic phenotype of blasts (red color) was characterized by the co-expression of CD33, CD14, CD15, CD64, HLA-DR, CD11c and MPO

The immunophenotypic analysis, performed on PB and bone marrow (BM) aspirate samples confirmed the morphological findings of the presence of two abnormal malignant populations. The diagnosis of CLL was confirmed by demonstration of expression of mature B-cell markers (CD19, CD22, CD20 dim), the co-expression of CD5 and CD23 and the absence of immaturemarkers, such as CD34, Terminal deoxynucleotidyltransferase (TdT) and CD10. Similarly, the diagnosis of AML (FAB-M5) was confirmed by demonstration of expression of myeloid markers (CD13, CD15, CD33, HLA-DR, MPO) and monocytic markers (CD4, CD11c, CD14, CD64) and positive for CD38 and CD56 but negative for CD34 and CD117 (Fig. 2).

**Fig. 3:**
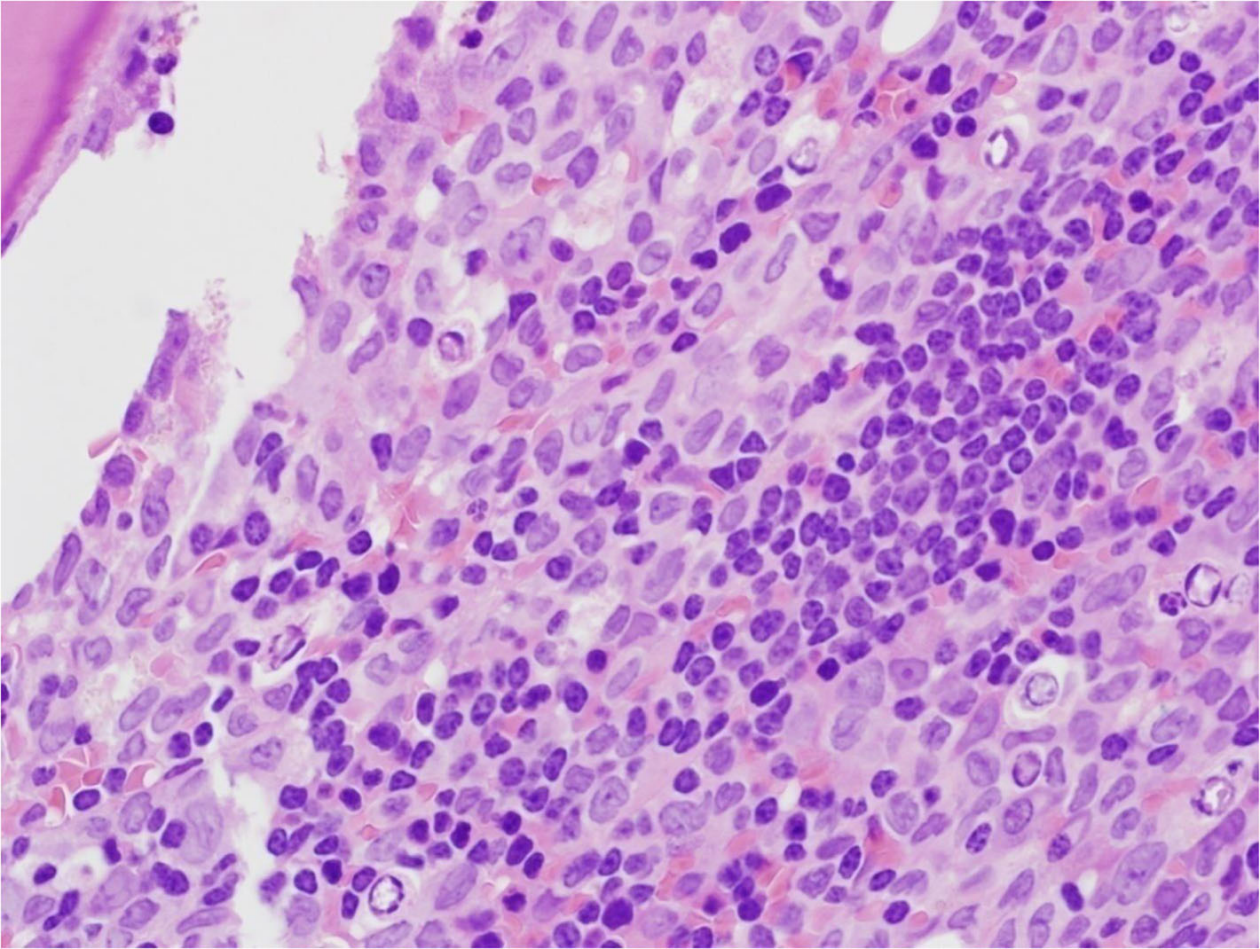
BM trephine biopsy infiltration by two discrete abnormal cell populations, one represents the leukemic blast cells with monocytoid features and the other represents small mature lymphocytes

**Fig. 4:**
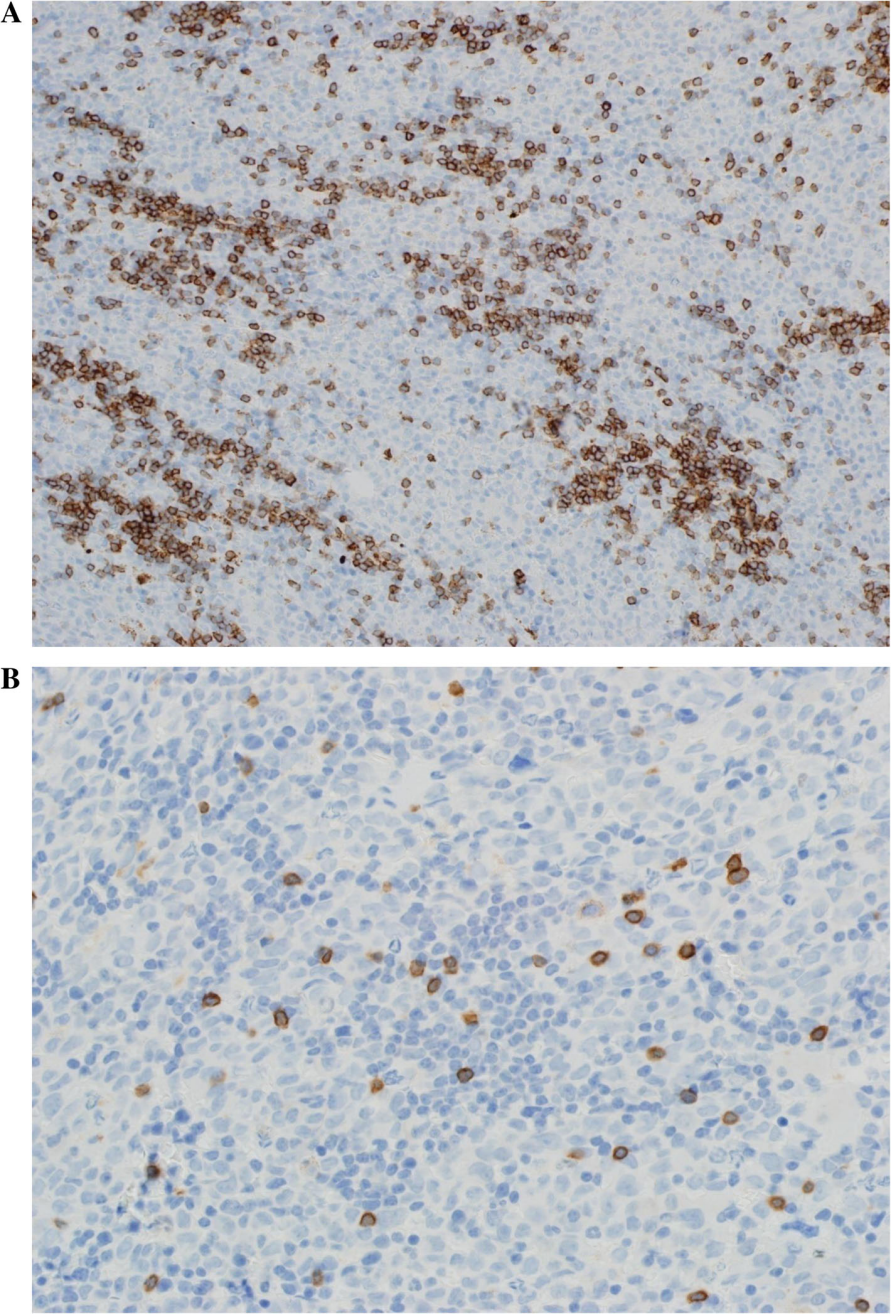
Immunohistochemical staining forl ymphocytes was positive for CD5 (**a**) and negative for CD3 (**b**)

**Fig. 5:**
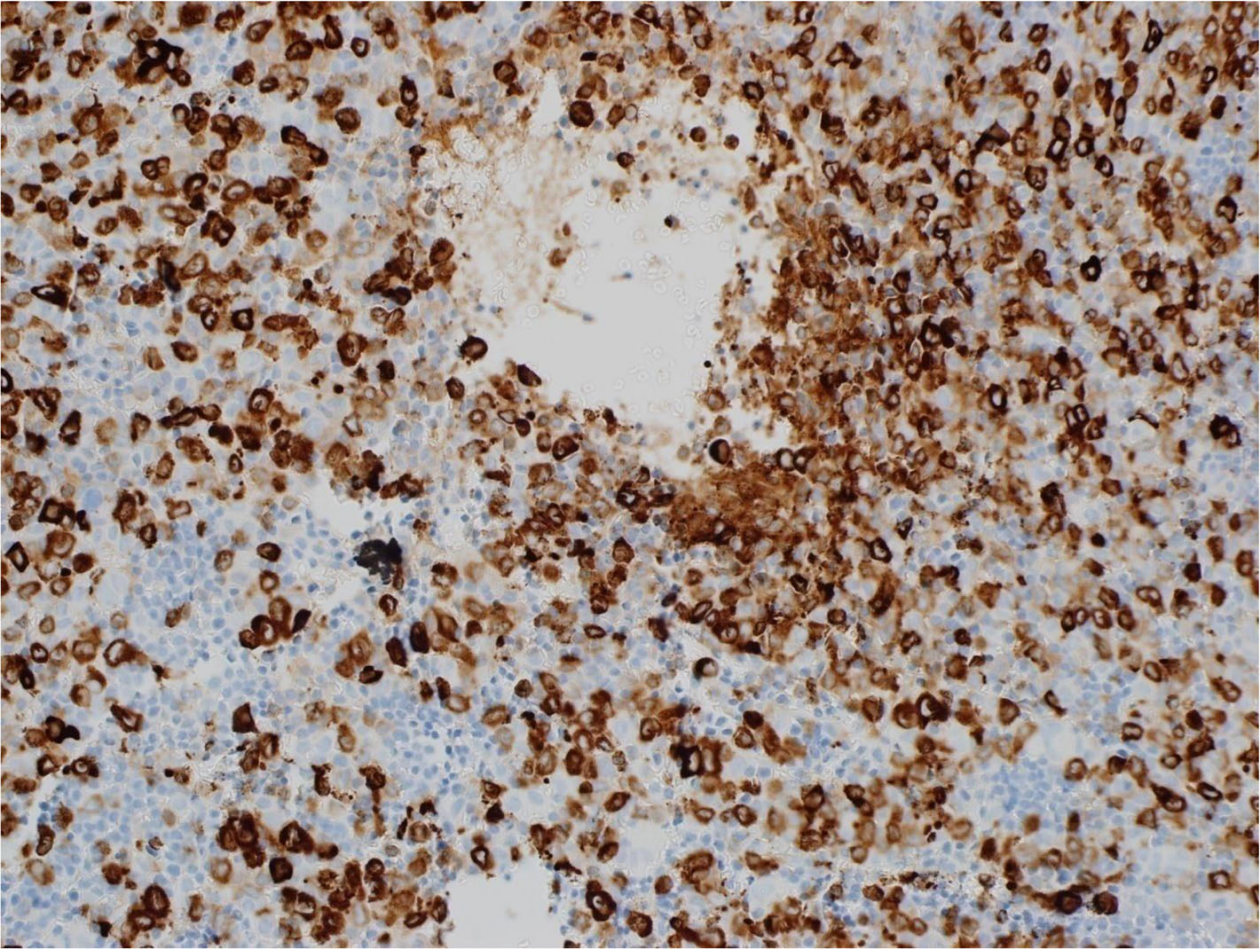
Immunohistochemical staining for blast cells was positive for MPO

The BM was hypercellular with a diffuse pattern of infiltration by intermixed lymphocytes and blasts similar to those observed in the PB (Fig. 3). The lymphocytes comprised approximately 30 % of the cellularity. Immunohistochemical staining for lymphocytes was positive for CD5, CD79a (not shown) and CD23 (not shown), negative for CD3 (Fig. 4). The blasts had monoblastic features, comprised approximately 60 % of BM cells and showed positivity byimmunohistochemistry (IHC) for MPO, CD43 (Fig. 5) and negative for CD3 and CD79a. The final diagnosis was reported as co-existence of CLL and AML with monoblastic features (FAB-M5).

On the basis of clinical features and *CBC* findings, the patient was started on hydroxyurea 1g three times a day, Allopurinol and intravenoushydration to prevent tumor lysis syndrome. His white blood cell count showed dramatic response within 7 days of treatment within the normal range (9.1 × 10^9^/L), Hydroxyurea was subsequently discontinued.

Later on, the kidney function tests started to deteriorate and allopurinol was changed to Rasburicase. He developed acute on top of chronic kidney disease and he was started on peritoneal dialysis.

Conventional cytogenetic analysis was performed on metaphase cells prepared from BM specimen cultured for 24 and 72 h without mitogens, using standard techniques. The result was reported as a normal karyotype male [46, XY]. Fluorescence in situ hybridization (FISH) analysis was performed for common abnormalities associated with CLL and AML according to the manufacturer's instructions. FISH analysis for CLL was performed using the following probes panel: TP53/CEP17, D13S319/13q34, IGH/CCND1 (DC,DF), IGH/BCL2 (DC,DF) whereas FISH analysis for AML was performed using the following probes panel: AML-ETO (DC,DF)/t(8;21)(q22;q22), PML-RARA (DC,DF)/t(15;17)(q22;q21), CBFB (DC,BAR)/inv(16)(p13;q22), MLL (DC,BAR)/11q23 and CEP8/D8Z2. FISH results were negative for both CLL and AML panel- specific abnormalities. RNA isolated from the BM sample according to the manufacturer's instructions was subjected to reverse transcriptase-polymerase chain reaction (RT-PCR) using a nested PCR reaction, to ascertain the presence or absence of AML specific abnormalities mentioned before. Similarly, DND was extracted according to the manufacturer’s instruction to detect both *FLT3 gene* mutations (ITD and D835) using published primers [13, 14] and reported negative for both AML specific abnormalities and *FLT3 gene* mutations. PCR amplifications of genomic DNA established the clonal nature of the B-cell expansion by the presence of the IgH gene rearrangement.

Due to old age, comorbid conditions and his performance status, the patient received palliative therapy with no curative treatment of his hematological malignancies. The patient died on the tenth day of hospitalization due to complications of both metabolic functions and infection.

The association of different malignancies with chronic lymphocytic leukemia has been reported. The most common second malignancies are solid tumors, especially lung and skin cancers [3]. The association of AML and CLL has been previously described. Most of these cases have been developed after treatment of CLL with chemotherapeutic agents suggesting that AML may be a secondary leukemia. Both disorders have been occasionally diagnosed simultaneously in untreated patients, appearing as two distinct and unrelated malignancies [3, 4].

In the present case study, the PB and BM morphologic findings are of AML with monoblastic features. FCM and IHC data are also consistent with AML-M5 but in addition, flow cytometric studies detect the presence of a lymphoproliferative process, CLL (CD5+/CD19+). The association of AML and CLL has been previously described [4–6]. Cytogenetic and FISH studies performed on interphase cells revealed no cytogenetic abnormalities such as deletions in chromosomes 6q21, 11q22, 13q14, and 17p13, and trisomy 12(+12) which are usually detected in 60–80 % of CLL patients [15, 16].

The history of the patient in the present study did not reveal any exposure to known leukemogenic agents or chemotherapy for CLL. The diagnoses of both AML and CLL were simultaneous, although we cannot rule out a previous asymptomatic indolent course of CLL. Although the simultaneous occurrence of AML and CLL is rare, it should not be overlooked as a possible cause of lymphocytosis in AML patients, as it has been reported that a reactive polyclonal lymphocytosis may occur in isolated cases of AML or, more commonly, in myelodysplastic syndrome patients [17].

Multiple theories behind the development of simultaneous malignancies in patients with CLL have been proposed [11]. The first theory involves immunosuppression reported in these patients [18]. Some authors hypothesize that the simultaneous occurrence of AML and CLL may be due to a common stem cell defect or leukemogenic factors or possibly a genetic susceptibility in some patients [19]. However, concomitant AML and CLL occurring due to chance cannot be excluded [20]. In treatment-related cases, in addition to decreased immune competence, the cytotoxicity and DNA damage induced by chemotherapy are also contributing factors [1, 5, 21]. However, others have demonstrated that the phenomenon results from separate karyotype abnormalities in the myeloid and lymphoid lines, triggering two separate neoplastic events [6, 11, 22–24]. Our findings support the idea that this rare concurrence of AML and untreated CLL may represent two separate disease processes as CLL arises from abnormal population of CD5+, CD19+ B lymphocytes whereas AML derives from an abnormal myeloid precursor.

## Conclusions

In summary, we have reported a case in which there was simultaneous presentation of AML and CLL. Both forms of leukemia were well documented by morphology, cytometric analysis and molecular studies. This increases the number of cases in which concomitant CLL and AML have been found without previous exposure to a cytotoxic agent or irradiation. Our findings support the idea that this rare concurrence of AML and untreated CLL may represent two separate disease processes.

## Abbreviations

AML: Acute myeloid leukemia
BM: Bone marrow
CLL: Chronic lymphocytic leukemia
CT: Computerized tomography
FAB: French-American-British
PB: Peripheral blood
PCR: Polymerase chain reaction
TdT: Terminal deoxynucleotidyltransferase
